# Medication Adherence in Adults with Chronic Diseases in Primary Healthcare: A Quality Improvement Project

**DOI:** 10.3390/nursrep14030129

**Published:** 2024-07-17

**Authors:** Claúdia Jorge Oliveira, Helena Maria Guerreiro José, Emília Isabel Martins Teixeira da Costa

**Affiliations:** 1 Health School, Polytechnic Institute of Beja, 7800 Beja, Portugal; 2 Health Sciences Research Unit: Nursing (UICISA: E), Nursing School of Coimbra (ESEnfC), 3046 Coimbra, Portugal; hjose@uatla.pt (H.M.G.J.); eicosta@ualg.pt (E.I.M.T.d.C.); 3 School of Health, University Institute Atlântica, 2730 Barcarena, Portugal; 4 Nursing Department, Health School, University of Algarve, 8000 Faro, Portugal

**Keywords:** clinical audit, chronic disease, evidence-based practice, health plan implementation, medication adherence, primary health care

## Abstract

(1) Background: Medication adherence is influenced by a variety of intricate factors, presenting hurdles for nurses working to improve it among adults with chronic conditions. Pinpointing the reasons for non-adherence is crucial for customizing interventions. The objective of this quality improvement project was to improve medication adherence among adults with chronic diseases in primary healthcare by promoting evidence-based practices, identifying barriers and facilitators to compliance, and developing strategies to ensure optimal adherence through engaging the nursing team, enhancing knowledge, and evaluating the effectiveness of the implemented strategies. (2) Methods: This study was a quality improvement project that utilized the JBI Evidence Implementation framework, the Practical Application of Clinical Evidence System, and the Getting Research into Practice audit tool across three phases: (i) forming a project team and conducting a baseline audit, (ii) offering feedback via the GRiP tool, and (iii) conducting a follow-up audit to assess best practice outcomes. The study was conducted between September 2021 and March 2022 in the community care unit of Algarve Regional Health Administration, targeting adults with chronic illnesses. (3) Results: A total of 148 individuals were audited, including 8 nurses, 70 baseline patients, and 70 post-implementation patients. Initial compliance with key best practices was low, with several criteria at 0% compliance at baseline. Post-intervention, we observed significant improvements; compliance with key best practices improved dramatically, with many reaching 100%. Notable improvements included enhanced patient education on medication management, regular medication adherence assessments, and increased engagement of healthcare professionals in adherence activities. (4) Conclusions: This quality improvement project demonstrated that structured, evidence-based interventions could significantly enhance medication adherence among adults with chronic diseases. The success of the project highlights the potential of similar strategies to be applied broadly in primary healthcare settings to improve health outcomes.

## 1. Introduction

Medication adherence refers to the extent to which individuals follow their prescribed medication regimen as directed by healthcare professionals [1,2]. It encompasses a complex interplay of factors involving patients, caregivers, healthcare providers, and health systems [3,4,5]. Suboptimal adherence to prescribed medications among those with chronic diseases poses a significant public health challenge, leading to adverse outcomes such as increased hospital admissions, reduced health improvements, heightened morbidity and mortality rates, and escalated healthcare expenses [1,2,6].

Medication adherence involves a multifaceted process where individuals align their behavior with healthcare provider recommendations, encompassing dosage, timing, frequency, and duration [5,7,8]. This process comprises three key steps: initiation (e.g., obtaining and taking the first dose), implementation (e.g., adhering to prescribed doses on schedule), and discontinuation (e.g., avoiding premature cessation) to ensure medication persistence [1,5].

Non-adherence to medication is influenced by various factors and manifests differently from person to person, posing a challenge for nurses. Understanding the underlying reasons for non-adherence is crucial for tailoring interventions aimed at promoting positive medication management behaviors and compliance. The World Health Organization delineates five dimensions of non-adherence, including social, economic, healthcare system, condition-related, therapy-related, and patient-related factors [1,5].

Of note are healthcare system and patient-related factors. Challenges within the healthcare system, such as limited access to healthcare professionals, medication coverage constraints, high costs, unclear instructions, and insufficient patient education materials, hinder medication adherence. Conversely, patient-related factors encompass unintentional lapses, especially prevalent with complex medication regimens, and intentional non-adherence driven by economic, belief, adverse effect, or expectation-related reasons [6].

Statistics reveal alarming rates of non-adherence, with approximately 50% of patients failing to adhere to prescribed medications and up to 10% of hospital readmissions attributed to medication non-adherence [2,5]. In the United States, roughly half of prescribed medications are taken incorrectly, primarily due to errors in dosage, frequency, timing, and duration, with around 20% of new prescriptions going unfilled [3,6]. Suboptimal medication adherence results in unfavorable treatment outcomes, dose mismanagement, heightened health risks, misdiagnoses, increased healthcare utilization, hospitalizations, and costs [1,2,6].

Given the prevalence and adverse impact of suboptimal medication adherence, urgent action is warranted to support individuals with chronic diseases requiring long-term medication regimens [1,5,9]. Enhancing medication adherence is imperative and recognized as a public health priority, with potential to alleviate the health and economic burdens associated with chronic conditions [6].

Evidence suggests that adherence rates can be improved through systematic incorporation of multiple strategies across the continuum of nursing care. Cost-effective interventions, such as utilizing blister packs and pillboxes to simplify medication regimens, have demonstrated efficacy in reducing unintentional non-adherence [2,5]. Moreover, team-based care interventions, encompassing medication tailoring, patient education, collaborative care, and voice messaging, have shown significant enhancements in adherence rates [6,10].

Advancements in health information technology offer promising avenues for improving medication adherence by providing real-time insights into medication usage and chronic condition management [6]. However, the sustainability of intervention benefits over time remains a concern, emphasizing the need for ongoing research and standardized methodologies to ensure long-term effectiveness [11].

Addressing health literacy disparities, especially among vulnerable populations, is vital for optimizing medication adherence interventions. Tailoring patient education materials and communication strategies to accommodate diverse health literacy levels, cultural backgrounds, and language preferences enhances intervention effectiveness [5,6].

To facilitate evidence translation into practice, a comprehensive approach is warranted, encompassing access to healthcare professionals, patient empowerment through education, reduction of medication access barriers, and utilization of health information technology tools for informed decision-making [5,6].

Despite the available evidence, gaps persist in the integration of evidence-based strategies into clinical practice, underscoring the need for targeted interventions. Recognizing this need, a project was initiated in the community care unit of Algarve Regional Health Administration (ARSA), Portugal, to enhance medication adherence among adults with chronic diseases. This project, undertaken by a multidisciplinary team, aimed to improve patient care outcomes and overall health by addressing medication management challenges effectively.

## 2. Objective

The objective of this quality improvement project was to improve medication adherence among adults with chronic diseases in primary healthcare by promoting evidence-based practices, identifying barriers and facilitators to compliance, and developing strategies to ensure optimal adherence through engaging the nursing team, enhancing knowledge, and evaluating the effectiveness of the implemented strategies.

## 3. Materials and Methods

This quality improvement project used the JBI Evidence Implementation framework [12], the software JBI Practical Application of Clinical Evidence System (PACES) version 2022 (Joanna Briggs Institute, Adelaide, Australia), and the Getting Research into Practice (GRiP) audit and feedback tool [13].The JBI Implementation approach is grounded in the audit and feedback process along with a structured approach to the identification and management of barriers to compliance with recommended clinical practices. It consists of seven stages: (1) identification of practice area for change, (2) engaging change agents, (3) assessment of context and readiness to change, (4) review of practice against evidence-based audit criteria, (5) implementation of changes to practice, (6) re-assessment of practice using a follow-up audit, and (7) consideration of the sustainability of practice changes.

The project was implemented in a primary healthcare setting that offers at-home nursing care in Portugal.

This quality improvement project involved three phases:

(1) Establishment of a project team and undertaking a baseline audit informed by the evidence;
(2) Reflection on baseline audit results to design and implement strategies;
(3) Completion of a follow-up audit (18 March 2022) to assess the outcomes of the intervention implemented to improve practice and identification of future practice issues to be addressed in subsequent audits.

This project was officially recognized as a Quality Improvement Activity by the Community Care Unit and received the endorsement of the coordinating body. Given its classification as a quality improvement initiative, formal ethical approval from the Ethics Committee of ARS Algarve was not required. However, the project team diligently followed ethical research standards, safeguarding participant confidentiality, anonymity, and their right to opt out at any point. These actions reflect our dedication to maintaining the utmost ethical standards throughout the project’s execution.

Following the project’s endorsement as a Quality Improvement Activity by the Community Care Unit, it is key to recognize that such initiatives do not require formal ethical approval like traditional research. Quality improvement projects focus on enhancing existing healthcare practices with minimal participant risk, hence facing less stringent regulatory demands. Nonetheless, our team strictly adhered to ethical principles including confidentiality, respect for individuals, and justice. Internal review processes ensured these standards were consistently maintained, demonstrating our commitment to ethical excellence throughout the project.

## 4. Procedure

### 4.1. Phase 1: Stakeholder Engagement (or Team Establishment) and Baseline Audit

The first phase of the project focused on forming the project team and conducting an initial audit based on evidence-informed criteria. A central group of essential stakeholders, including researchers, specialist nurses, and registered nurses, was gathered to support the project’s aims. The project leader managed the development and oversight of the project, while the team members engaged in various tasks such as gathering data, providing feedback, and helping to implement strategies aimed at improving medication adherence in adults with chronic conditions. Team members, each bringing their own valuable expertise and knowledge, played roles that included support, data gathering, data entry, and active participation. The team was led by a coordinator, who was both a researcher and specialist nurse, tasked with overseeing the project coordination, audit management, and training. The co-coordinator, also a researcher, was tasked with strategy design and methodological support. Other team members included an additional researcher, a head nurse who served as a clinical facilitator, and two registered nurses responsible for data collection, designing educational materials, and applying research into practice. Together, this multidisciplinary group collaboratively conducted the initial and subsequent audits, using their collective skills to boost medication adherence. This phase of the quality improvement project was conducted from September to November 2021.

The entire nursing team underwent formal training on promoting medication adherence among adults with chronic diseases based on evidence-based practices. Monthly video calls were scheduled for all team members to discuss implementation phases. Meeting dates and project documentation were communicated via email.

The primary goal of the baseline audit was to assess the variance between current practices and best practices supported by evidence. Table 1 outlines the audit criteria to be employed during both the baseline and follow-up audits, providing a description of the sample for each criterion and measures of compliance. These audit criteria are derived from the JBI Evidence Summary titled “Adults with chronic diseases: Medication adherence strategies” [13].

Inclusion criteria for patient participants were established to ensure a focused and relevant sample for the study and were consistently applied across all phases. Eligible participants were adults with diagnosed chronic illnesses who were actively receiving care from the project’s primary healthcare team. Additionally, all participants were required to be on a prescribed medication regimen, with the minimum requirement of taking at least one medication regularly. Information was gathered through a questionnaire. The baseline audit was scheduled for November 2021.

### 4.2. Phase 2: Design and Implementation of Strategies to Improve Practice (GRiP)

This phase aimed to identify barriers contributing to the disparity between current practices and the best practices identified in the baseline audit. Additionally, the team developed strategies to encourage the adoption of best practices. This phase of the quality improvement project took place from November 2021 to December 2021.

Using the JBI-PACES software, version 2022 (Joanna Briggs Institute, Adelaide, Australia), all the project team members analyzed the results of the baseline audit and collaborated on strategies to enhance adherence to best practices for medication adherence among adults with chronic diseases in community healthcare settings.

The JBI-GRiP tool was utilized to record all identified barriers, strategies, and required resources for instigating change. Furthermore, we engaged key stakeholders, elicited feedback, and fostered adaptation through small group sessions conducted via Zoom due to the limitations imposed by the COVID-19 pandemic.

The first meeting took place on 18 November 2021 with the project team (coordinator, co-coordinator, researcher, head nurse, and two RNs). The purpose of this meeting was to present the results of the baseline audit and to discuss and analyze the possible barriers and proposed strategies to overcome them.

The second meeting, which took place on 10 December 2021, was held with the coordinator, head nurse, and the two RNs.

Following the meeting, an email containing the audit results and strategies was distributed to the remaining team members. This approach was necessary due to the challenges of convening everyone in person, which were compounded by the pandemic and the geographic dispersion of some team members. The implementation of changes, along with the necessary explanatory and training logistics for the team, took place from December 2021 through February 2022.

### 4.3. Phase 3: Follow-Up Audit Post-Implementation of Change Strategy

The purpose of the follow-up audit was to evaluate any improvements in adherence to best practices and to pinpoint areas that still needed further refinement and improvement. Conducted on 18 March 2022, the follow-up audit utilized the same evidence-based audit criteria employed in the baseline audit. Data from the follow-up audit were inputted into the JBI-PACES system. A comparative analysis was then performed between the baseline and follow-up audit data to evaluate any changes in compliance rates.

**Figure 1:**
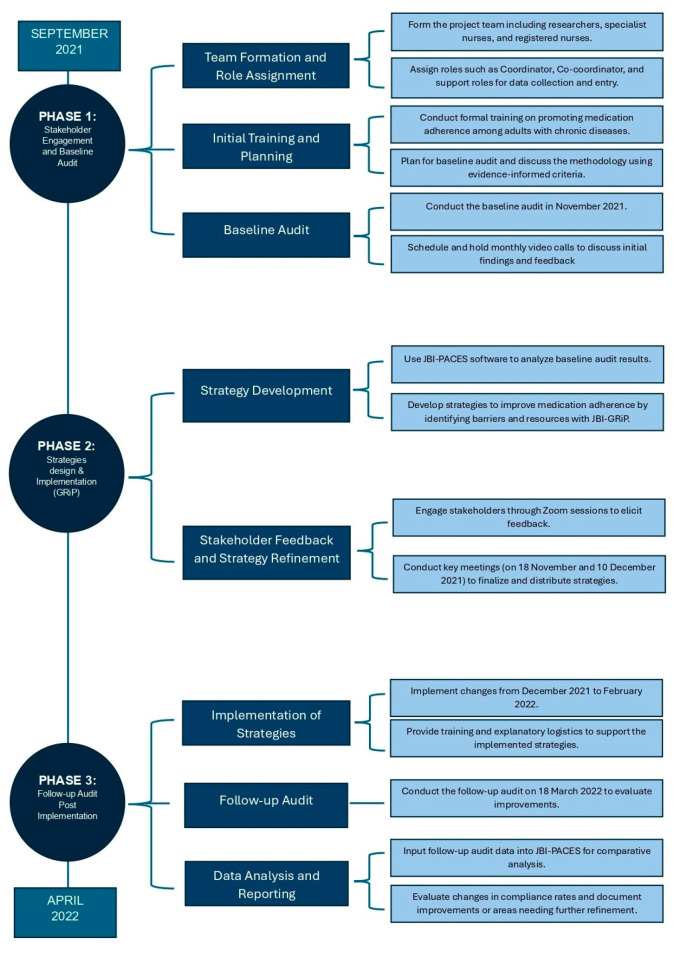
Flowchart of quality improvement project phases.

Figure 1 presents a flowchart that outlines all phases of the quality improvement project.

### 4.4. Analysis

This project was undertaken within a specific Community Care Unit of ARSA, a region where challenges related to medication adherence among adult and elderly individuals with chronic diseases had been identified. Recognizing the critical importance of addressing this issue, we adopted a comprehensive mixed-method approach to thoroughly assess and evaluate our intervention strategies.

Direct observation emerged as a crucial tool in our evaluation process. By closely observing the practices of nurses within the unit, we were able to gain valuable insights into their adherence to evidence-based best practices concerning the promotion of medication adherence. This hands-on approach allowed us to directly assess the implementation of our intervention strategies and identify areas for improvement.

Building upon the findings from our baseline data analysis, we developed targeted interventions aimed at addressing the identified barriers to medication adherence. These interventions were carefully tailored to the specific needs and challenges observed within the community care unit.

Following the implementation of our intervention strategies, we collected post-intervention data using the same criteria as in the baseline assessment. This enabled us to measure the impact of our interventions and evaluate any changes in medication adherence practices among both nurses and patients.

To determine the statistical significance of our study results, rigorous statistical analysis was conducted. This involved analyzing the data collected before and after the implementation of our interventions to assess any notable differences and ascertain the effectiveness of our intervention strategies.

Overall, our project delved deep into the complexities of medication adherence within the community care setting, employing a multifaceted approach to address this critical issue and ultimately improve patient outcomes.

## 5. Results

A total of 148 people were audited: 8 nurses, 70 patients from the baseline, and 70 patients from the post-implementation period. The average age of the baseline sample was 78.74 (SD = 6.177), and in the post-implementation period it was 75.88 (SD = 7.264). At the baseline, 64.3% of the patients were female and 35.7% were male. After the best practice implementation, 58.6% of the patients were female, and 41.4% were male.

The Section 5 is organized in alignment with the three phases outlined in the Section 3.

### 5.1. Phase 1: Baseline Audit

**Figure 2:**
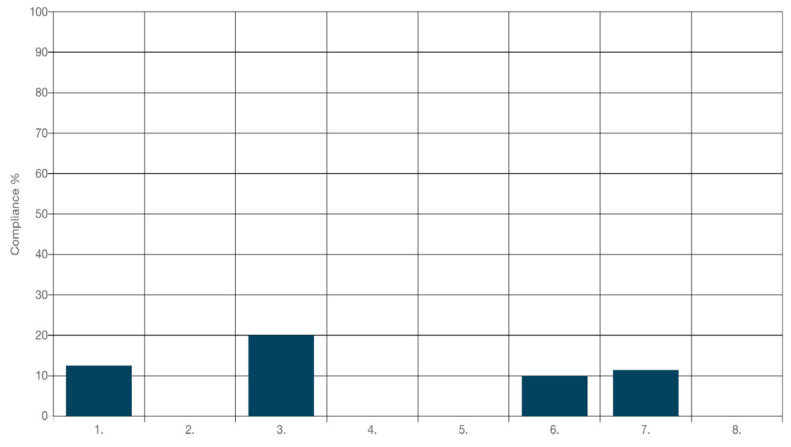
Compliance (%) with best practice for audit criteria (1–8) of medication adherence in adults with chronic diseases in primary healthcare at baseline (November 2021).

Based on the baseline audit results processed through the JBI-PACES software, it became evident that compliance with best practices (Audit Criteria 1 to 8) for medication adherence among adults with chronic diseases in primary healthcare was notably deficient, as illustrated in Figure 2. This analysis highlighted critical gaps in care delivery, pinpointing specific areas where urgent improvements were necessary. Notably, criteria No. 2, No. 4, No. 5, and No. 8 (see Table 1) were completely unmet during the baseline audit, signaling significant lapses in the nursing team’s implementation of these essential best practices. The lack of adherence to established guidelines, as identified through the systematic analysis facilitated by the JBI-PACES software, raises serious concerns about the quality and effectiveness of interventions aimed at enhancing medication adherence in this vulnerable patient population. Addressing these deficiencies and ensuring comprehensive adherence to best practices, as mapped and tracked using the JBI-PACES software, is crucial for optimizing patient outcomes and enhancing the overall quality of care in primary healthcare settings.

### 5.2. Phase 2: Strategies for Getting Research into Practice (GRiP)

After meticulous analysis of the baseline results, a total of nine barriers were discerned, shedding light on the complexities inherent in achieving optimal medication adherence among adults with chronic diseases in primary healthcare settings. These barriers encapsulate a spectrum of challenges ranging from resource constraints to systemic inefficiencies, each posing a unique hurdle to effective patient care.

Identifying these barriers is a crucial initial step toward creating targeted interventions to overcome these challenges and enhance adherence rates. Importantly, Table 2 was compiled during Phase 2, following a meticulous analysis of the baseline audit results. It details the specific and common strategies used, the resources required, and the anticipated outcomes for each strategy. This comprehensive overview acted as a guide for healthcare practitioners, allowing them to understand the complexities of medication adherence and implement significant changes that positively affected patient health outcomes.

### 5.3. Phase 3: Follow-Up Audit

**Figure 3:**
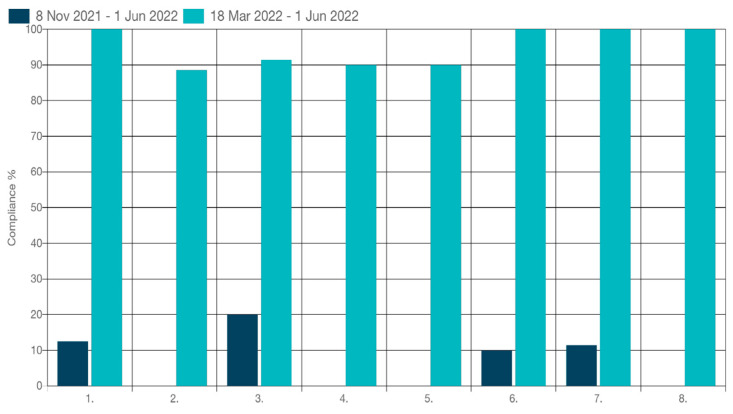
Compliance (%) with best practice for audit criteria (1 to 8) of medication adherence in adults with chronic diseases in primary healthcare at baseline (November 2021) and follow-up cycle (March 2022).

Upon completion of the implementation period for the strategies aimed at promoting medication adherence among elderly individuals with chronic diseases, a final audit was conducted. It is worth mentioning that this audit adhered to the same methodology and criteria previously defined by JBI, in line with best practices in this area. As illustrated in Figure 3, significant improvements were achieved across all criteria (see Table 1), with criteria 1, 6, 7, and 8 reaching a compliance rate of 100%. Criterion 2 (Adults with chronic diseases are assessed for medication adherence by healthcare professionals in the following components: assessing medication adherence, readiness to change, lifestyle factors, patient treatment goals, barriers to adherence, side effects or persistent symptoms, and level of literacy) obtained the lowest compliance rate (88.57%); however, this was considered quite positive by the researchers.

## 6. Discussion

In this study, the aim was to contribute to the promotion of evidence-based practice regarding medication adherence among adults with chronic diseases receiving nursing care at home in Portugal’s primary healthcare setting. The methodology involved conducting baseline and follow-up audits, with a total sample size of 148 individuals, including 8 nurses and 140 patients (70 from the baseline and 70 from the follow-up audit). The study was conducted in the primary healthcare facilities where interventions were implemented. The choice of this context was based on a person-centered practice approach, advocating for nursing practice characterized by closeness, providing care in the comfort and safety of the individual’s home.

The quality improvement project achieved significant improvements in compliance with best practices regarding nursing interventions to promote medication adherence among adults with chronic diseases in Portugal’s primary healthcare setting. In the baseline audit, compliance rates were remarkably low, potentially impacting medication adherence rates negatively. According to several studies, initially, approximately 20–30% of new prescriptions were not filled [14,15,16]. Recent systematic reviews comparing initiation rates across various chronic diseases found non-initiation rates of about 10% to 25% [17]. A study conducted in Portugal demonstrated that older people with diabetes did not adhere to their medications as prescribed, but after the implementation of a complex intervention delivered by nurses, medication adherence rates significantly increased [18]. In this context, the discussion emphasizes the pivotal role of nursing in promoting medication adherence.

Through meticulous analysis during Phase 2, several key barriers to medication adherence were identified, such as a lack of knowledge about best practices, nonexistent guidelines, and insufficient skills among healthcare providers. To address these challenges, comprehensive strategies were developed and implemented, including training and support via formal and informal educational sessions, the development of comprehensive procedures, and enhancing communication between patients and healthcare professionals. These aspects have also been emphasized by other authors [19].

Training sessions were aimed at improving healthcare providers’ understanding and ability to apply best practices, while comprehensive procedures and protocols were established to standardize care across the board. Additionally, the emphasis on improving communication skills in healthcare settings aligns with demographic shifts and the growing prevalence of chronic diseases, underlining the importance of a person-centered practice approach that advocates for nursing care characterized by closeness, providing care in the comfort and safety of the individual’s home.

The study’s methodology, involving baseline and follow-up audits, revealed that prior to interventions, adherence rates were significantly low, mirroring broader trends noted in the literature where a notable percentage of prescriptions were initially unfilled (14–18). However, post-implementation, the compliance rates for essential criteria showed substantial improvement, highlighting the effectiveness of the strategies employed. The project thus not only addressed immediate gaps in medication adherence but also contributed to a broader understanding of implementing person-centered, evidence-based practices effectively in community settings to enhance healthcare outcomes. This comprehensive approach has proven crucial in overcoming the barriers identified and enhancing the overall quality of care provided in primary healthcare settings.

Aligned with demographic shifts, the population is aging, leading to a growing prevalence of at least one chronic disease diagnosis. In this regard, it is undeniable that adults and older people require adherence to medication regimens, which are often complex. However, it is crucial to recognize the complications associated with non-adherence as well as the predictive factors for it. Non-adherence consequences include deterioration of the condition, increased prevalence of comorbid diseases, heightened healthcare expenses, and mortality [20,21,22,23].

As previously mentioned, the WHO has outlined five main categories of factors influencing medication adherence: (i) condition-related, (ii) therapy-related, (iii) socio-economic, (iv) healthcare- and system-related, and (v) patient-specific factors [16,24].

Given the nature and methodology of the current project implementing best practices, we will focus our discussion on factors related to healthcare professionals and the healthcare system. Research suggests that long waiting times and non-directed and short-duration consultations may contribute to non-adherence [25,26,27,28]. Additionally, better communication between patients and healthcare professionals, particularly establishing a therapeutic relationship centered on the individual, promotes adherent behaviors [29,30,31,32,33,34].

In this same vein, the literature emphasizes the importance of shared decision-making, where nursing consultations facilitate dialogue between the patient and nurse, making the patient feel that the therapeutic plan is based on the best scientific evidence, the nurse’s knowledge, and their own preferences. In this regard, studies show that patients who actively participate in the decision-making process about their treatment are more likely to adhere to treatment compared to those who do not [35,36,37,38].

According to a study conducted by Zoromski and Frazier [39], nurses have the responsibility and capacity to assist individuals with chronic diseases in managing their medication regimens through a person-centered intervention, integrating their technical-scientific knowledge with the individual’s preferences, beliefs, and attitudes. According to the same authors, medication adherence can be promoted if nurses develop a complex, individualized, and systematic intervention based on best practices [39]. Therefore, nurses should intervene collaboratively with other healthcare professionals, possess technical and scientific knowledge to convey this knowledge to the individual/family, and be able to implement behavioral and educational interventions [39]. Educational interventions are essential as they enhance health literacy, making individuals more aware of the risks associated with ineffective medication management.

There is no doubt that the non-adherence to medicines is a global problem compromising health and economic outcomes for individuals and society [5]. The main headline results reveal significant improvements in medication adherence practices following the implementation of targeted strategies. Compliance rates for criteria No. 1, No. 6, No. 7, and No. 8 reached 100%, marking a notable enhancement compared to the baseline findings. However, criterion No. 2 exhibited a lower compliance rate of 88.57%, albeit showing positive progress from the baseline. Analyzing the criterion with the lowest compliance rate, it can be considered that the complexity of the criterion itself may have been the reason for the lower adherence rate [40,41]. In this sense, measures will be implemented aimed at achieving it, probably through formal and informal training sessions.

The relative utility and impact of the practice change strategies employed were substantial, as evidenced by the notable improvements observed across all audit criteria. The facilitators and interventions planned and reported in GRiP played a pivotal role in driving these positive changes [12]. The discussion meticulously links the specific resources utilized to support each strategy, elucidating the interventions undertaken to address the identified barriers and effectuate improvements.

Despite the significant findings, this study has several limitations that merit consideration. First, the relatively small sample size and the study’s geographic limitation to primary healthcare settings in Portugal may restrict the generalizability of the results to wider populations or different healthcare systems. Additionally, while the study achieved improvements in medication adherence, the short duration of follow-up audits may not fully capture long-term adherence outcomes or the sustainability of the implemented interventions. Another limitation is the potential variability in the intervention’s application due to differences in individual nurse practices, which might affect the consistency of the results. Lastly, the exclusion of certain demographic variables in our analysis, such as socio-economic status or educational level, could have prevented deeper insights into the barriers to medication adherence. Future studies could address these gaps by incorporating a more extensive range of variables and a longer follow-up period to better understand the long-term impact of the interventions.

## 7. Conclusions

While effective strategies and facilitators have been highlighted, it is imperative to acknowledge any inefficacious approaches. These findings contribute valuable insights into understanding which strategies are effective in diverse contexts, guiding future implementation efforts.

Key lessons drawn from this project include the importance of comprehensive planning, stakeholder engagement, and tailored interventions to address identified barriers. By situating the findings within the broader literature, this study underscores the significance of context-specific interventions in promoting medication adherence.

Looking ahead, sustaining improvements necessitates ongoing monitoring, adaptation, and integration of strategies into routine practice. Plans for future sustainability should encompass continued education, system-wide support, and proactive management of identified barriers to ensure enduring positive outcomes.

## Figures and Tables

**Table 1:** Audit criteria, sample and method employed to measure compliance with strategies to promote medication adherence in adults with chronic diseases (JBI criteria).

Audit Criteria (JBI Criteria)	Sample	Method Used to Measure % Compliance with Best Practice
Nurses, alone or in collaboration with other healthcare professionals, are engaged to assist adults with chronic diseases to improve their medication adherence post-discharge.	Nurses Baseline *n* = 8 Follow-up *n* = 8	The nurses of the project team checked via a questionnaire their engagement to assist adults with chronic diseases to improve their medication adherence. If the nurse completed the questionnaire with a score equal to or higher than 90%, the auditor would mark a “Yes”. If the nurse obtained a score lower than 90%, the auditor would mark a “No”.
2. Adults with chronic diseases are assessed for medication adherence by healthcare professionals in the following components: assessing medication adherence, readiness to change, lifestyle factors, patient treatment goals, barriers to adherence, side effects or persistent symptoms, and level of literacy.	Patient interview Baseline *n* = 70 Follow-up *n* = 70	Nursing staff would mark as follows: “Yes” if the patient/family had received the information about medication management from the nurse. “No” if the patient/family had not received the information about medication management from the nurse. “N/A” if the patient/family was not sure whether he/she had received the information about medication management.
3. Adults with chronic diseases are assessed for medication adherence on a regular basis.	Review patient case notes Baseline *n* = 70 Follow-up *n* = 70	Nursing staff would mark as follows: “Yes” if the patient was assessed for medication adherence on a regular basis. “No” if the patient was not assessed for medication adherence on a regular basis.
4. Adults with chronic diseases receive education from healthcare professionals on the following components: instructions on how to take their prescribed medication (verbal and written), what do in the case of missed doses, the health condition for which the medication is prescribed, the consequence of non-adherence, and the therapeutic benefits of effective medication adherence.	Patient interview Baseline *n* = 70 Follow-up *n* = 70	Nursing staff would mark as follows: “Yes” if the patient/family had received the education from the healthcare professional. “No” if the patient/family had not received the education from the healthcare professional. “N/A” if the patient/family was not sure whether he/she had received the education from the healthcare professional.
5. Where appropriate, cognitive behavioral therapy and/or patient psychoeducation is provided to those with mental health conditions to reduce negative perceptions regarding medication-taking.	Review patient case notes Baseline *n* = 30 Follow-up *n* = 30	Nursing staff would mark as follows: “Yes” if the patient was assessed for cognitive behavioral therapy and/or patient psychoeducation provided. “No” if the patient was not assessed for cognitive behavioral therapy and/or patient psychoeducation provided.
6. Adults with chronic diseases receive strategies from healthcare professionals to change their behavior around medication adherence.	Patient interview Baseline *n* = 70 Follow-up *n* = 70	Nursing staff would mark as follows: “Yes” if the patient/family had received the strategies from healthcare professionals to change their behavior around medication adherence. “No” if the patient/family had not received the strategies from healthcare professionals to change their behavior around medication adherence. “N/A” if the patient/family was not sure whether he/she had received the strategies from healthcare professionals to change their behavior around medication adherence.
7. Strategies to improve medication adherence in adults with chronic diseases are delivered face-to-face.	Observation Baseline *n* = 70 Follow-up *n* = 70	Nursing staff would mark as follows: “Yes” if the strategies to improve medication adherence in adults with chronic diseases were delivered face-to-face. “No” if the strategies to improve medication adherence in adults with chronic diseases were not delivered face-to-face.
8. Family or other support networks of adults with chronic diseases are engaged by healthcare professionals to help improve medication adherence.	Patient interview Baseline *n* = 70 Follow-up *n* = 70	Nursing staff would mark as follows: “Yes” if the family or other support networks of adults with chronic diseases were engaged by healthcare professionals to help improve medication adherence. “No” if the family or other support networks of adults with chronic diseases were not engaged by healthcare professionals to help improve medication adherence. “N/A” if the family or other support networks of adults with chronic diseases were not sure if they were engaged by healthcare professionals to help improve medication adherence.

**Table 2:** Approaches to overcome barriers: strategies, resources, and outcomes.

Barrier	Strategy	Resources	Outcomes
Lack of knowledge about best practice concerning strategies to improve long-term medication adherence	Training and support through formal and informal educational sessions	Team meeting E-mail information	Improve the level of knowledge about best practice concerning strategies to promote medication adherence
	Provide written information to the team (e.g., pamphlets)	Written information for professionals Written information for professionals (on-paper and computer support)	Increase in compliance with documented strategies to promote medication adherence
Nonexistent guidelines on following medication instructions	Development of a comprehensive procedure for promoting medication adherence among adults with chronic disease	Team meeting E-mail information Protocol/flowchart to promote medication adherence	Increase in compliance with recommendations
Low documentation about medication adherence	Provide scientific paper related to best practice concerning strategies to increase medication adherence	Team meeting Written information for professionals (on-paper and computer support)	Increase in compliance with recommendations
Insufficient skills/capacity to implement	Audit, review, and feedback the results	Team meeting E-mail information	Involve the nurse team to change behavior to increase compliance with recommendations
Low compliance with care plans related to medication adherence and events of non-adherence	Training and support via formal educational sessions	Team meeting E-mail information	Increase in compliance with care plans related to medication adherence
	Provide written information about the care plans (e.g., elaboration of flowchart)	Written information for professionals (paper and e-mail)	
Strength of habits and difficulty in changing	Availability to explain issues	Meetings	Involve the nurse team to change behavior to increase compliance with recommendations
	Regular reminders of the new mode of care	E-mail information	
	Limit number of changes at the same time	Team meeting by negotiation	
Perception of an individual project	Insist on promoting quality of care	Team meeting	Ensure that nurses are involved and motivated to increase compliance with recommendations
	Involve all the nurses in the implementation of the best practice	Team meeting	
	Disseminate the results among members of the nurse team	Team working	
Organizational barriers	Support of the leader	Head nurses have to review the distribution of workloads	Ensure that nurses are motivated to increase compliance with recommendations
Lack of human resources	Explain to the team about the different approach, including the time factor	Team meeting Head nurses	Ensure that nurses are motivated to increase compliance with recommendations
	Promote multidisciplinary meeting to increase medication adherence	Multidisciplinary team meeting	

## Data Availability

The data presented in this study are available on request from the corresponding author. The data are not publicly available due to privacy restrictions.

## Public Involvement Statement

There was no public involvement in any aspect of this research.

## Guidelines and Standards Statement

This manuscript was drafted against the SQUIRE2.0 (Standards for Quality Improvement Reporting Excellence) [43] for Quality Improvement studies.
